# Spectroscopic and Structural Characterization of Reduced *Desulfovibrio vulgaris* Hildenborough W-FdhAB Reveals Stable Metal Coordination during Catalysis

**DOI:** 10.1021/acschembio.2c00336

**Published:** 2022-06-29

**Authors:** Ana Rita Oliveira, Cristiano Mota, Kateryna Klymanska, Frédéric Biaso, Maria João Romão, Bruno Guigliarelli, Inês Cardoso Pereira

**Affiliations:** † Instituto de Tecnologia Química e Biológica António Xavier, Universidade Nova de Lisboa, Av. da República, 2780-157 Oeiras, Portugal; ‡ Associate Laboratory i4HB—Institute for Health and Bioeconomy, NOVA School of Science and Technology, Universidade NOVA de Lisboa, 2829-516 Caparica, Portugal; § UCIBIO, Applied Molecular Biosciences Unit, Departament of Chemistry, NOVA School of Science and Technology, Universidade NOVA de Lisboa, 2829-516 Caparica, Portugal; ∥ Laboratoire de Bioénergétique et Ingénierie des Protéines, Aix Marseille Univ, CNRS, BIP, Marseille 13402, France

## Abstract

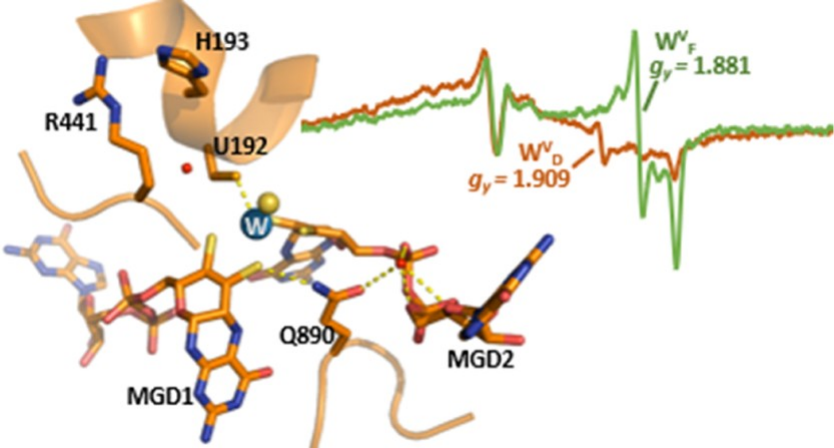

Metal-dependent formate dehydrogenases are important enzymes due to their activity of CO_2_ reduction to formate. The tungsten-containing FdhAB formate dehydrogenase from *Desulfovibrio vulgaris* Hildenborough is a good example displaying high activity, simple composition, and a notable structural and catalytic robustness. Here, we report the first spectroscopic redox characterization of FdhAB metal centers by EPR. Titration with dithionite or formate leads to reduction of three [4Fe–4S]^1+^ clusters, and full reduction requires Ti(III)–citrate. The redox potentials of the four [4Fe–4S]^1+^ centers range between −250 and −530 mV. Two distinct W^V^ signals were detected, W_D_^V^ and W_F_^V^, which differ in only the *g*_2_-value. This difference can be explained by small variations in the twist angle of the two pyranopterins, as determined through DFT calculations of model compounds. The redox potential of W^VI/V^ was determined to be −370 mV when reduced by dithionite and −340 mV when reduced by formate. The crystal structure of dithionite-reduced FdhAB was determined at high resolution (1.5 Å), revealing the same structural alterations as reported for the formate-reduced structure. These results corroborate a stable six-ligand W coordination in the catalytic intermediate W^V^ state of FdhAB.

## Introduction

Formate dehydrogenases (FDHs) are very important enzymes due to their ability to reduce carbon dioxide. The metal-dependent FDHs found in anaerobic prokaryotes present orders of magnitude higher activities for CO_2_ reduction than the metal-independent ones.^1,2^ Therefore, metal-dependent FDHs are interesting biocatalysts for technologies to mitigate the increasing levels of CO_2_, producing formate that is an attractive alternative green fuel and a hydrogen carrier.^3^ In these FDHs, the active site is well conserved with the metal being coordinated by four sulfur atoms from two molybdopterin guanosine dinucleotide (MGD) ligands, one labile sulfur and either a S from cysteine or a Se from selenocysteine.^2,4,5^ The active-site coordination with two MGDs is characteristic of the dimethyl sulfoxide reductase (DMSOR) family of Mo/W-bisMGD enzymes.^4−6^

Among metal-dependent FDHs, the tungsten-containing ones are particularly active for CO_2_ reduction.^7,8^ Tungsten is the heaviest element found in biological systems and the only one from the third transition row of the periodic table with an identified biological function.^9,10^ However, its use is limited to some bacteria and archaea, and in the case of some hyperthermophiles, it is even essential.^10,11^ Tungsten and molybdenum are chemically analogous, sharing similar properties, namely, the ionic radii, coordination chemistry, and redox properties.^9^ The diversity of W-enzymes is higher than initially expected, and tungsten seems to be the biological choice for some low-potential redox processes, such as CO_2_ reduction (by FDHs and formylmethanofuran dehydrogenases), carboxylic acid reduction [by aldehyde:ferredoxin oxidoreductases (AORs)], aromatic ring reduction (by benzoyl CoA-reductases), or acetylene hydration (by acetylene hydratases).^10−13^ Therefore, the biotechnological interest in W-enzymes has been growing.^13^

FDH was the first W-enzyme to be discovered, as the NAD^+^-dependent FDH from *Clostridium thermoaceticum*, revealing the biological relevance of W.^14^ W-containing FDHs have only been identified in obligatory anaerobes,^12^ with the *Methylobacterium extorquens* FDH as the only example of a W-dependent enzyme in aerobic bacteria.^15^ Initially, it was believed that only W-containing FDHs would be capable of CO_2_ reduction, due to the more negative redox potentials of W,^12^ but it is now known that Mo-FDHs can also reduce CO_2_.^16,17^ The FdhAB (Fdh-1) from the sulfate-reducing bacterium *Desulfovibrio vulgaris* Hildenborough is a good example of a W-dependent FDH, showing very high activity for CO_2_ reduction,^8^ which is only surpassed by more complex and very oxygen-sensitive FDHs.^7,16,18^ FdhAB has the great advantage of being tolerant to oxygen exposure in the oxidized state, allowing its purification and handling in air.^8^ Moreover, its simple composition with only two subunits and structural and catalytic robustness make this FDH an excellent model system for biocatalytic applications.^19−22^ The crystal structure of FdhAB was recently determined in both oxidized and formate-reduced states,^8^ providing important insights into the metal coordination in the fully reduced state. However, there is still no information on possible catalytic intermediates in this enzyme. Also, the spectroscopic characterization of W-enzymes is still scarce, when compared to Mo-enzymes,^23^ and this work aimed at characterizing FdhAB using EPR spectroscopy, focusing on W^V^ and reduced [4Fe–4S]^1+^ centers. In addition, the crystal structure of FdhAB reduced with dithionite was obtained at 1.5 Å, the highest resolution attained for an FDH reduced form so far. The spectroscopic and structural data obtained confirm a stable coordination of selenocysteine to the W, both in the fully reduced (W^IV^), oxidized (W^VI^), and intermediate W^V^ states of the enzyme, which is very relevant to elucidate the mechanism of CO_2_ reduction.

## Results and Discussion

### EPR Characterization of the As-Isolated Fdh

EPR analysis of dithionite-reduced FdhAB at 15 K revealed a rhombic signal with *g*-values at 2.042, 1.941, and 1.898 typical of reduced [4Fe–4S]^1+^ clusters (Figure S1Ba). This signal is superimposed on broad lateral lines with *g*-values around *g* = 2.06 and 1.86, exhibiting faster relaxation properties. The whole spectrum accounts for about 2.2 spin/molecule, and the broad lateral lines may arise from different FeS signals or may be due to intercenter spin–spin couplings.^24−26^ Upon increasing the temperature, these [4Fe–4S]^1+^ signals broaden progressively (Figure S1B). At 80 K, another rhombic signal at *g* = 1.990, 1.909, and 1.851, with slow relaxation properties, is easily detected, revealing the partial reduction of the W cofactor to the W^V^ state, as described below. This signal is termed W_D_^V^.

When the protein is reduced by excess formate, similar [4Fe–4S]^1+^ signals are observed, accounting for up to 2.9 spin/molecule (Figure S1C), but in this case, a different W^V^ signal is observed, which we named W_F_^V^, which shows a small shift in *g*_2_ to 1.88. In order to analyze the redox properties of the metal centers, we performed EPR titrations of FdhAB using either dithionite or formate as the reductant.

### Redox Titration with Dithionite

**Figure 1:**
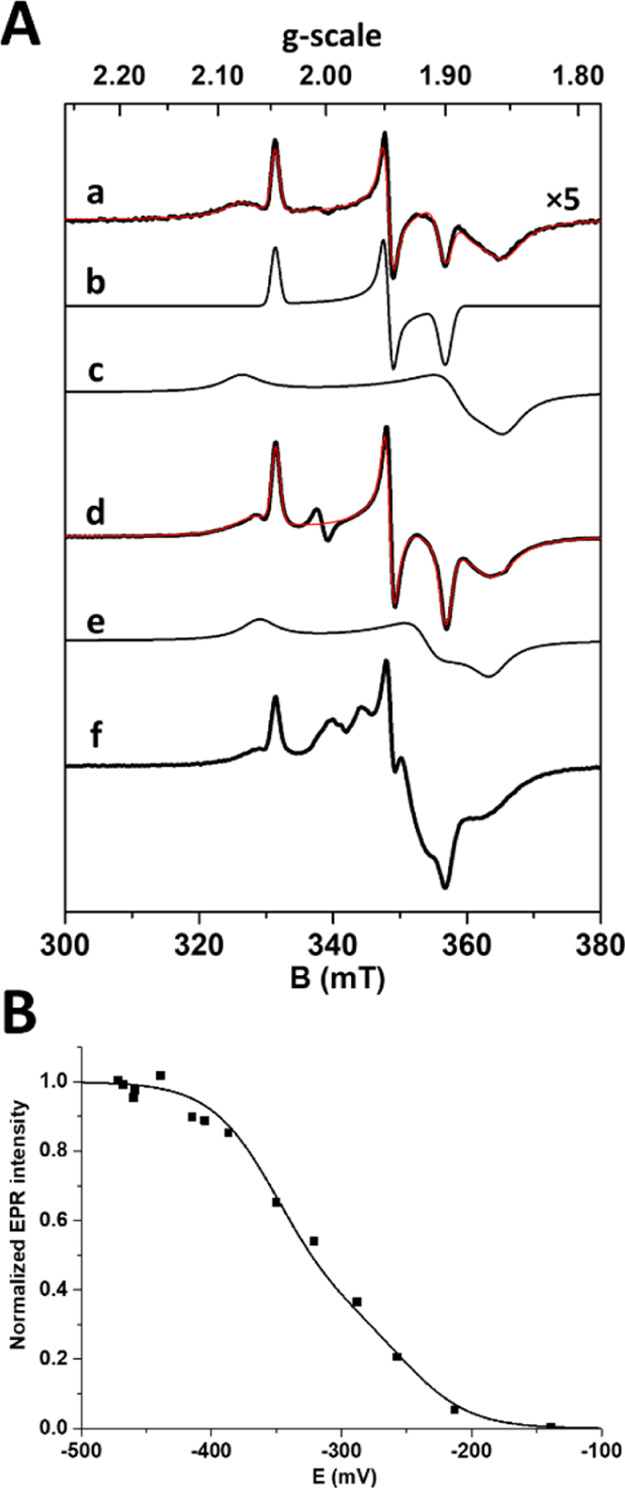
EPR spectra of dithionite-reduced FdhAB. (A) Samples poised at −257 (a) and −439 mV (d), superimposed with simulations (red) made by the addition of spectral components FeS1 (b), FeS2 (c), and FeS3 (e) (parameters in Table 1). (f) Sample reduced with Ti(III)–citrate (−525 mV) after subtraction of the Ti(III)–citrate signal. Temperature, 15 K; microwave power, 1 mW at 9.481 GHz; and modulation amplitude, 1 mT at 100 kHz. (B) Redox potential dependence of the FeS signal intensity, fitted with two Nernst processes with *E*°1 = −250 mV and *E*°2 = −350 mV, 1:2 relative contribution.

A titration was performed at pH = 7.6 by small additions of dithionite, in the presence of redox mediators. A progressive increase in the EPR signals typical of reduced [4Fe–4S]^1+^ clusters was observed below −200 mV (Figure 1A and Table 1). Both previously observed rhombic components increase simultaneously, exhibiting different relaxation properties, indicating that at least two clusters undergo reduction in the same potential range. Spectral simulations show that at −257 mV, the spectrum is well described by superimposition of two rhombic FeS signals, FeS_1_ and FeS_2_, with an intensity ratio of 1:3 (Figure 1Aa). The simultaneously reduced [4Fe–4S]^1+^ centers exhibit markedly different *g*-tensor anisotropy, line width, and relaxation properties as observed in other FDHs.^27,28^ Below −320 mV, a third broad FeS_3_ component is required to account for the EPR spectral shapes (Figure 1Ad). Spin intensity measurements show that maximal intensity is reached at about −450 mV and corresponds to 2.6 (±10%) spin/molecule, indicating that one cluster is not reduced in the potential range investigated. The variation of signal intensity as a function of potential is well described by the superimposition of two independent one-electron processes centered at −250 and −350 mV (±10 mV) with approximately 1:2 relative contributions (Figure 1B). Interestingly, the amplitude variations of the different [4Fe–4S]^1+^ signal components follow approximately the same behavior (Figure S2), suggesting that some redox cooperativity between the [4Fe–4S]^1+^ centers likely occurs, as observed in other complex FeS enzymes.^29,30^

**Table 1:** EPR Parameters of FeS Signals Used for Spectral Simulations (Figure 1A)^a^

				*E* = −257 mV		*E* = −439 mV	
	*g*_1_	*g*_2_	*g*_3_	relative proportions	spin intensity	relative proportions	spin intensity
FeS_1_	2.044(1) (0.0090)	1.944(1) (0.0075)	1.898(1) (0.0110)	1	0.13	1	0.7
FeS_2_	2.075(2) (4.2)	1.889(2) (4.2)	1.853(2) (4.2)	3	0.39	1	0.7
FeS_3_	2.059(2) (3.7)	1.914(2) (3.7)	1.863(2) (3.7)	0	0	1.7	1.2
total spin intensity					0.52		2.6

a Spectral broadening was described by the *g*-strain effect for FeS1 (parameters in brackets), while for FeS2 and FeS3, isotropic Lorentzian broadening is considered (line widths in mT given in the second bracket). Spin intensity of the different signals are given in spin/molecule.

**Figure 2:**
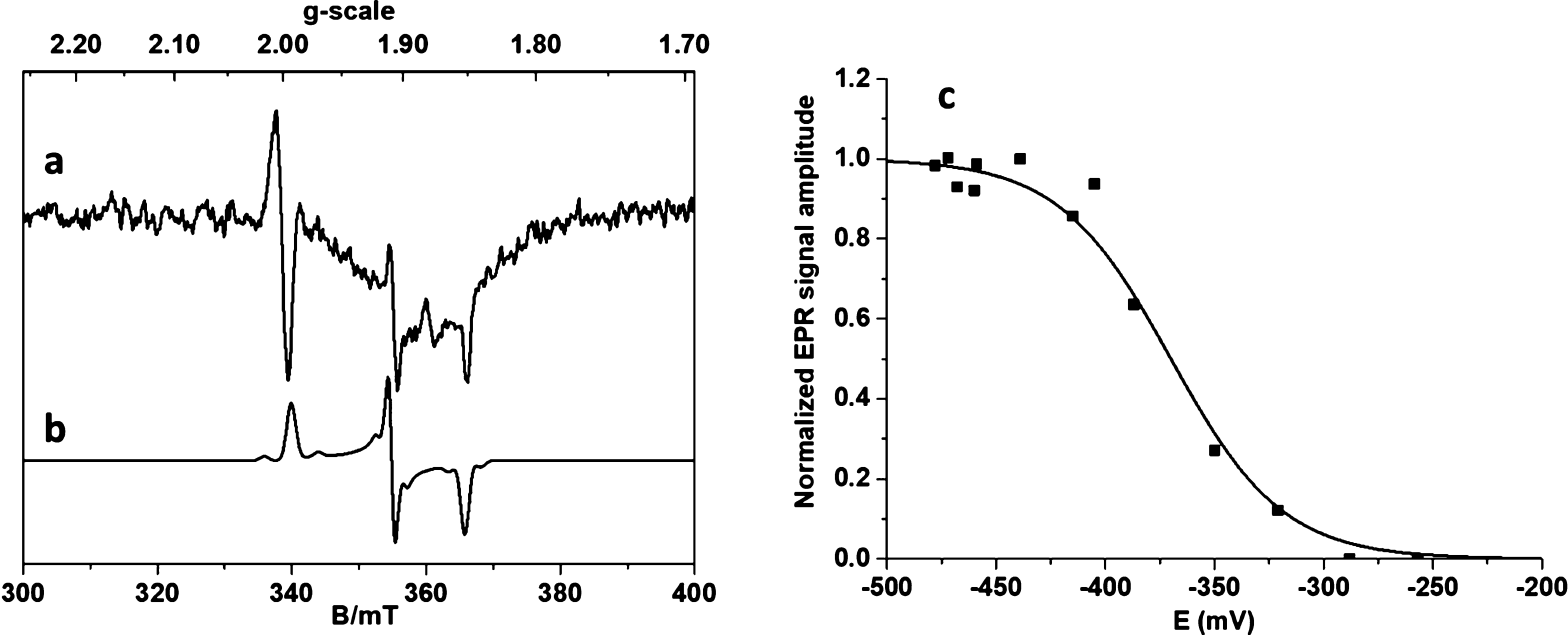
EPR signal of dithionite-reduced FdhAB (W_D_^V^). (a) Experimental spectrum. Conditions: redox potential, −439 mV; temperature, 80 K; microwave power 40 mW; and modulation amplitude, 1 mT. (b) Simulation with parameters from Table 2. (c) Redox behavior of the W_D_^V^ signal, measured at *g*_3_ = 1.852, fitted with a Nernst process with *E*° = −370 mV.

When the reduced samples are analyzed at temperatures >70 K, a W^V^ signal (W_D_^V^) appears below −300 mV, where only the major components at *g*_2_ = 1.909 and *g*_3_ = 1.852 can be observed, while *g*_1_ is hidden by the mediator radical signal (Figure 2a). The *g*-values and line widths of this signal are reminiscent of those found for other W-enzymes^23^ (Figure 2b). An additional minor derivative line is observed around *g* = 1.88, indicating that the W^V^ signal is slightly heterogeneous. The W^VI^/W^V^ reduction follows the Nernst law with a midpoint potential of −370 mV (±10 mV) (Figure 2c). The maximum W_D_^V^ signal intensity is about 0.02 spin/molecule, indicating that only a minor proportion of the W cofactor is reduced by dithionite.

### Redox Titration with Formate

**Figure 3:**
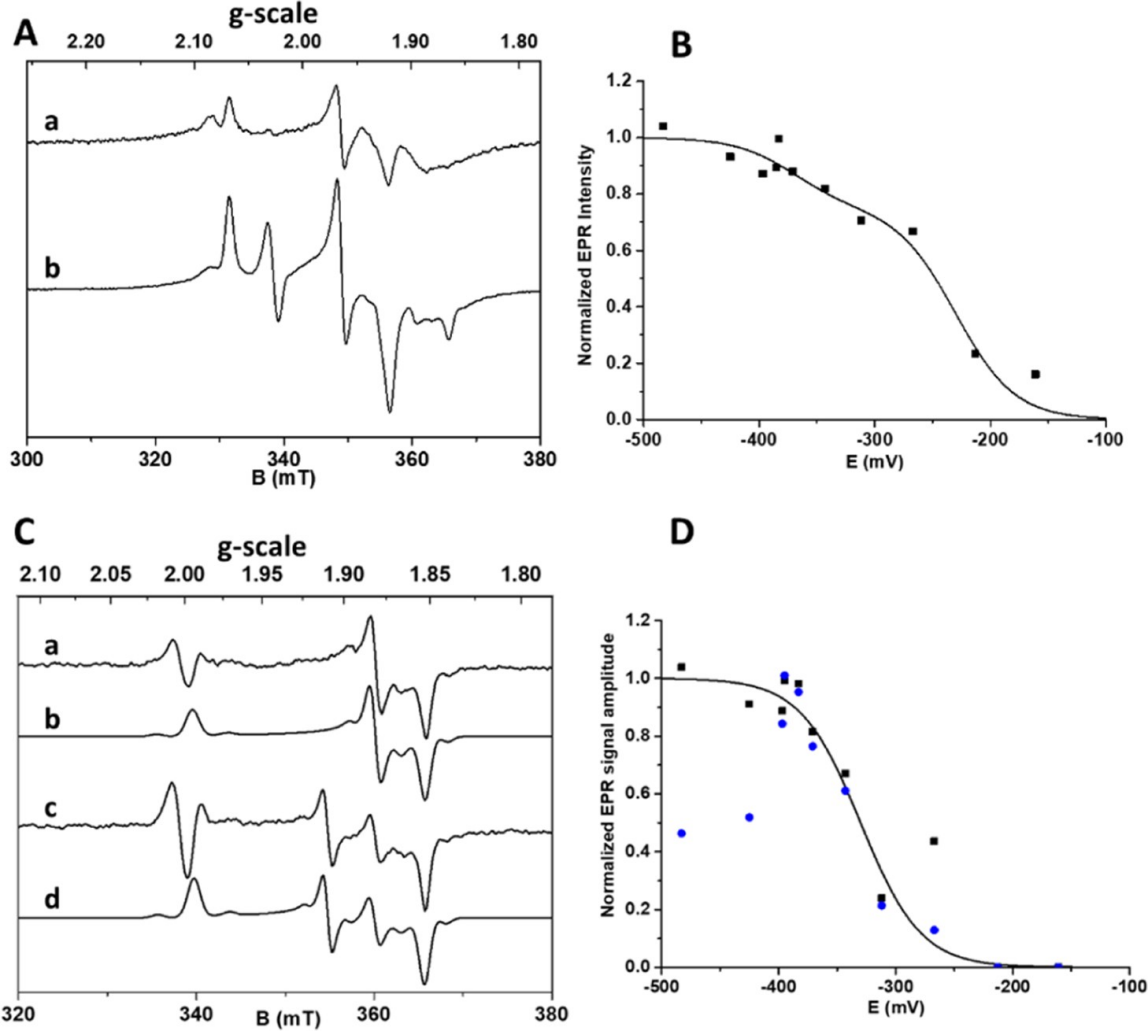
EPR redox titration of FdhAB with formate. (A) Samples at 15 K and (a) −213 and (b) −483 mV. (B) Variation of the [4Fe–4S]^1+^ signal intensity, fitted with two Nernst processes at *E*°1 = −230 mV and *E*°2 = −370 mV, with 3:1 relative contribution. Conditions as in Figure 1C. (C) Samples at 80 K and (a) −395 mV after reduction with formate, (b) its simulation with only W_F_^V^ species, (c) −483 mV after reduction by formate and dithionite, and (d) its simulation with the W_D_^V^ and W_F_^V^ species with a 2:1 ratio (parameters in Table 2). EPR conditions as in Figure 2. (D) Amplitude variation of the *g* = 1.85 peak (black squares) represents all W^V^ species, fitted with a Nernst process centered at *E*° = −330 mV. Amplitude variation of the *g* = 1.88 line (blue circles) corresponds to the W_F_^V^ species only. The two samples poised <−400 mV were obtained by a subsequent addition of dithionite after formate.

In the second approach, dithiothreitol (DTT)-activated FdhAB was titrated by formate at pH = 7.6. DTT leads to some reduction of the [4Fe–4S]^1+^ centers (0.32 spin/molecule) (Figure 3Aa and Figure S1Ac), but no W^V^ signal is observed at 80 K. At the beginning of the reduction, the composite FeS spectrum is similar to that observed in the dithionite titration with only small differences in relative amplitude and line widths of spectral components. Upon stepwise reduction with formate, the complex [4Fe–4S]^1+^ signal increases progressively to reach about 1.9 spin/molecule around −400 mV. To reach lower redox potentials, dithionite was finally added, resulting in a moderate increase in the [4Fe–4S]^1+^ signal reaching 2.2 spin/molecule at −483 mV with no significant changes in the spectral shape (Figure 3A,b). The variation of the [4Fe–4S]^1+^ signal intensity is well described by the sum of two Nernst processes centered at −230 and −370 mV with 3:1 relative contribution (Figure 3B). This behavior is different from that observed with dithionite and indicates that the redox properties of the [4Fe–4S]^2+/1+^ centers are affected by the nature of the reductant.

Interestingly, upon formate reduction, a different and much more intense W^V^ species is observed (W_F_^V^), below −250 mV, characterized by a g_2_ of 1.881 (Figure 3C,D and Table 2). Its maximal intensity is around 0.2 spin/molecule, which is about 10-fold higher than that observed with dithionite. Dithionite was added to the most reduced sample treated with formate to reach a lower potential. Surprisingly, while the [4Fe–4S]^1+^ signal increases slightly to reach 2.2 spin/molecule (Figure 3A,B), the total spin intensity of the W^V^ species is not significantly modified (Figure 3D), but about 55–60% of the W_F_^V^ transitions to the W_D_^V^ species (Figure 3C). By taking the difference between both spectra, it was possible to obtain the signatures for each W^V^ species and to determine more accurately the magnetic parameters used for their simulation (Table 2). With formate, the redox potential of W^VI/V^ shifts to *E*° = −330 mV.

**Table 2:** EPR Parameters Used for Simulations of the W^V^ Signals^a^

	*g*_1_	*g*_2_	*g*_3_	*g*-anisotropy	*g*-rhombicity
W_D_^V^	**1.993** (0.009)	**1.909** (0.005)	**1.852** (0.007)	0.141	0.59
W_F_^V^	**1.995** (0.010)	**1.881** (0.006)	**1.852** (0.007)	0.143	0.79
^183^W hyperfine parameters	A_1_	A_2_	A_3_		
^183^W_D_	225 (20)	112(10)	123 (10)		
^183^W_F_	225 (20)	129(5)	134 (5)		

a Spectral broadening was described by the *g*-strain effect (parameters in brackets). Anisotropy (*g*_1_–*g*_3_) and rhombicity [(*g*_1_ – *g*_2_)/(*g*_1_ – *g*_3_)] were also calculated. The natural abundance of the isotope ^183^W (*I* = 1/2) was considered (14.3%), and hyperfine coupling parameters are given in MHz.

The presence of small lateral lines in W_D_^V^ and W_F_^V^, arising from hyperfine coupling with ^183^W (*I* = 1/2), is similar to those found for other tungstoenzymes.^10,23^ The magnetic parameters and redox potentials *E*°(W^VI/V^) are notably close to those determined for the W^V^ species of *Pyrococcus furiosus* AOR (*E*°(W^VI/V^) = −365 mV and *E*°(W^V/IV^) = −436 mV). As expected, the values determined for W in FdhAB are more negative than the ones determined for Mo in the *Desulfovibrio desulfuricans* Mo-FDH (*E*°(Mo^VI/V^) = −160 mV and *E*°(Mo^V/IV^) = −330 mV).^31^ In FdhAB, no decrease in the W^V^ signals was observed upon a further decrease in the redox potential, indicating that the W^V/IV^ redox transition occurs at potentials <−500 mV. Hence, the substoichiometric amount of W^V^ species is likely not due to close values of *E*°(W^VI/V^) and *E*°(W^V/IV^) but more likely arises from the presence of different W species at the active site. Such substoichiometry and heterogeneity of the W^V^ or Mo^V^ EPR species are commonly observed in Mo/W-bisMGD enzymes.^23,32−35^ In FdhAB, the strong increase in the W^V^ species observed after enzyme activation and reduction by its substrate supports the likely involvement of these species in the catalytic mechanism.

### Reduction with Titanium(III)–Citrate

As commonly observed with other FDHs,^27,36,37^ full reduction of FdhAB could not be achieved with dithionite or formate at neutral pH. Ti(III)–citrate was then used as a stronger reductant.^38^ The as-isolated FdhAB was reduced using a substoichiometric amount of Ti(III)–citrate. At 15 K, a sample poised at −420 mV revealed the same [4Fe–4S]^1+^ signal as observed at this potential with dithionite (Figure S3a). Similarly, this sample exhibited only a weak W_D_^V^ signal at 80 K. To reach potentials lower than −500 mV, an excess of Ti(III)–citrate had to be used, but in this case, the EPR signal of Ti(III) superimposes those of the enzyme. As the Ti(III) species has a much slower relaxation, its spectral contribution can be obtained at 80 K (Figure S3d). The [4Fe–4S]^1+^ signal at −525 mV could then be obtained by subtracting the Ti(III) contribution from the whole sample 15 K spectrum. The double integration of the obtained spectrum (Figure 1Af) indicates that it corresponds to 3.6 ± 0.3 spin/molecule, which is in agreement with the presence of four [4Fe–4S] centers in the enzyme, and shows that at this potential, the reduction of the FeS clusters is nearly complete. The reduction of the fourth cluster with Ti(III)–citrate leads to the appearance of additional features around *g* = 2.0, 1.96, and 1.92 and to the broadening of the lateral lines at 2.07 and 1.86, which reveals clearly the contribution of spin–spin coupling between several [4Fe–4S]^1+^ centers, as observed in other oxidoreductases.^25,26,29^ This enables us to estimate the [4Fe–4S]^1+^ center lowest midpoint potential as around −530 mV. In contrast to some FDHs,^27,39^ no EPR signals were detected at low field and high *g*-values, indicating the absence of high-spin (*S* > 1/2) FeS centers, consistent with the full Cys coordination of the four [4Fe–4S]^2+/1+^ centers as observed by crystallographic data.

In some of the Mo/W-bisMGD enzymes, magnetic couplings between the reduced proximal FeS center and the Mo/W^V^ species have been detected.^32,39,40^ In FdhAB, although the W^V^ species were detected at redox potentials where a high proportion of FeS clusters is reduced, no splitting due to intercenter coupling was observed. Spin–spin coupling could be weakened by a longer distance between the W cofactor and the nearest center (12.5 Å) or compensation between dipolar and exchange interactions, and its spectral effects may be hidden by the line width of the W^V^ signal.^40^ Another possibility is that the closest [4Fe4S] center is the cluster with the lowest potential, and the detection of magnetic interactions would require to measure the W^V^ signals at potentials much lower than −500 mV.

Overall, the redox titrations of FdhAB showed that the redox potentials of three FeS clusters are in the range between −230 and −370 mV with redox properties that are slightly dependent on the reductant used or on the activation process. The fourth FeS center has a significantly lower potential, below −500 mV, but globally, these redox potential values are in the range found for [4Fe–4S]^2+/1+^ clusters in multicenter FeS oxidoreductases such as nitrate reductase,^29^ pyruvate ferredoxin oxidoreductase,^25^ or hydrogenases.^30^ According to the value of the midpoint potential of the CO_2_/formate couple close to −440 mV at pH 7.6,^41^ the presence of an FeS cluster with a potential of about 100 mV lower seems to be unfavorable for the enzyme functioning. However, such a situation where the potential of an FeS cluster is out of the range between those of the electron donor and acceptor is not rare among oxidoreductases.^29,30^ For instance, in nitrate reductase NarGHI, there are two [4Fe–4S] clusters with potentials of −400 and −200 mV in the electron transfer chain, while the redox potential of the electron donor (menaquinone) is around −80 mV and of the electron acceptor (nitrate) is +420 mV.^29^ Such out-of-range potentials seem to have no influence on the electron transfer rate and enzyme activity and could be compensated by redox cooperativity (or anticooperativity) effects as proposed in some multicenter enzymes.^30^

### Dithionite-Reduced Crystal Structure

The crystal structures of FdhAB in the as-isolated and formate-reduced states were recently reported.^8^ Before this, the only reduced structure of an FDH was that of *Escherichia coli* FdhH,^42^ for which the X-ray data were later reinterpreted.^43^ The two interpretations differ mainly in the position of the selenocysteine and the neighboring amino acids. Although in the first structure, the Se is coordinating the Mo in the active site,^42^ in the second, the Se is dissociated from the metal and the whole loop is moved away 12 Å.^43^ One possible explanation for these differences may be the presence of mixed states in the crystal, raising doubts about the catalytic relevant form of the active site. Both the as-isolated and formate-reduced FdhAB structures showed Se from Sec192 bound to the W, with four sulfurs from the two MGDs and a sulfido ligand (W–SH/=S) completing the W coordination sphere. Given the different W^V^ species observed with dithionite and formate, we tried to elucidate possible structural changes occurring upon dithionite reduction. Crystals of the as-isolated enzyme were soaked with dithionite inside an anaerobic chamber, mimicking the conditions of the EPR titration. The resulting crystals (DvFdhAB_dith, PDB_code_7Z5O) diffracted beyond 1.5 Å, and the structure was solved by molecular replacement using the formate-reduced structure (PDB_code_6SDV). The model was refined to final crystallographic *R*_work_ and *R*_free_ values of 12.1 and 15.3% (see Table S1), respectively. The overall refined structure is identical to that previously determined from crystals soaked with formate, with RMSDs of 0.21 Å for 963 α-carbons (out of 978) in the catalytic subunit and 0.25 Å for 214 α-carbons (out of 236) in the small subunit. These differences increase slightly to 0.34 and 0.30 Å for the large and small subunits, respectively, when compared to the as-isolated structure (PDB_code_6SDR) (SuperPose, version 1.0; http://superpose.wishartlab.com/).

**Figure 4:**
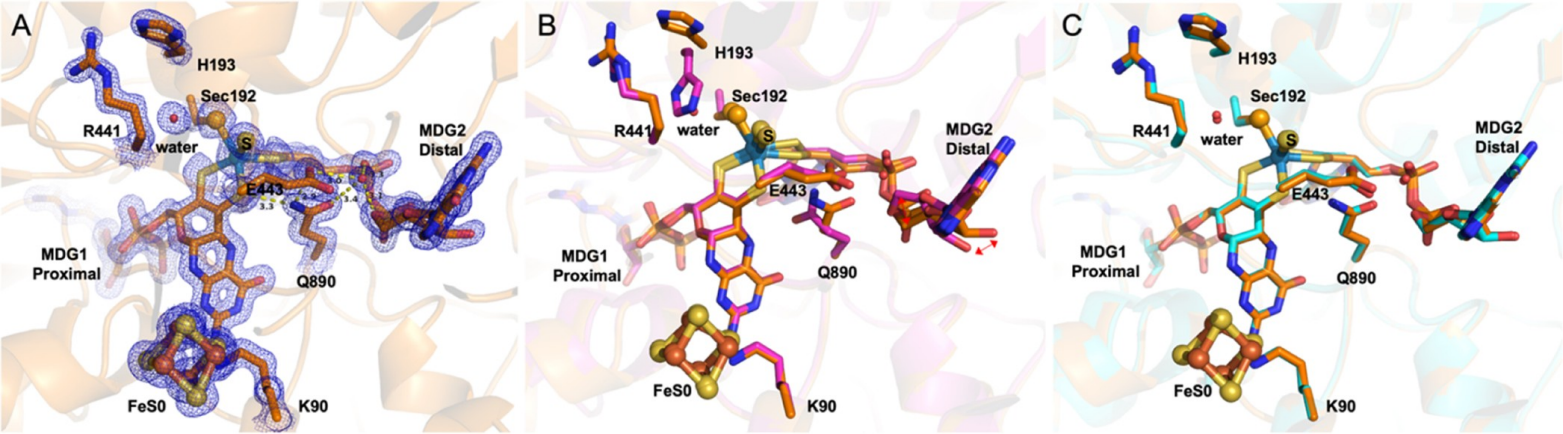
FdhAB active site. (A) Close-up view of the active site of FdhAB reduced with dithionite (colored by element with carbon in orange (PDB_code_7Z5O)). In blue mesh, the 2Fo-Fc map contoured at 1.5σ. (B) Superposition of the active sites of oxidized (colored by element with carbon in magenta (PDB_code_6SDR)) and dithionite-reduced structures. (C) Superposition of the active sites of formate-reduced (colored by element with carbon in cyan (PDB_code_6SDV)) and dithionite-reduced structures.

The high-resolution data collected allowed us to model the structure of the dithionite-reduced form with very well-defined electron density maps (Figure 4A). Superpositions of the active sites with the previous structures show that the proximal MGD-1, involved in electron transfer between the W and FeS0 cluster, is in the same position in the three conditions (Figure 4B,C). The electron transfer pathway likely involves Lys90 and a conserved water molecule, a feature observed in other members of the DMSOR family.^35^ On the other hand, in both the dithionite- and formate-reduced structures, the distal MGD-2, which is believed to modulate the redox behavior of the W, suffers a distortion in the ribose moiety, when compared with the as-isolated form (Figure 4B). Consistently, the MGD-2 conformational change is thought to be triggered by the rotamer orientation of the Gln890 side chain in the reduced state (Figure 4C).

Furthermore, analogous to formate, dithionite reduction leads to a small movement of SeCys and its corresponding loop, with Cα-SeCys192 shifting up about 1 Å and the His193 side chain moving away from the metal and being replaced by a water molecule. The adjacent α-helix (Ser194-Pro198) is also displaced up to 2.3 Å (measured at Cα-Thr196). Similar to the formate-reduced form, the catalytic metal site was refined with an occupancy of 1 and very reasonable *B*-factors (*W* = 14.9 Å^2^, *S*_12/1101_ = 15.0 Å^2^, *S*_13/1101_ = 15.6 Å^2^, *S*_12/1102_ = 15.1 Å^2^, *S*_13/1102_ = 15.1 Å^2^, Se = 18.9 Å^2^, and *S* = 17.5 Å^2^).

Overall, this structure is nearly identical to the formate-reduced one previously reported,^8^ which confirms full reduction by formate. Additionally, it reveals that equivalent forms are obtained by crystallization regardless of the reductant, whereas in solution, small differences were observed by EPR for the intermediate W^V^ species.

### Molecular Modeling of W^V^ Species

**Figure 5:**
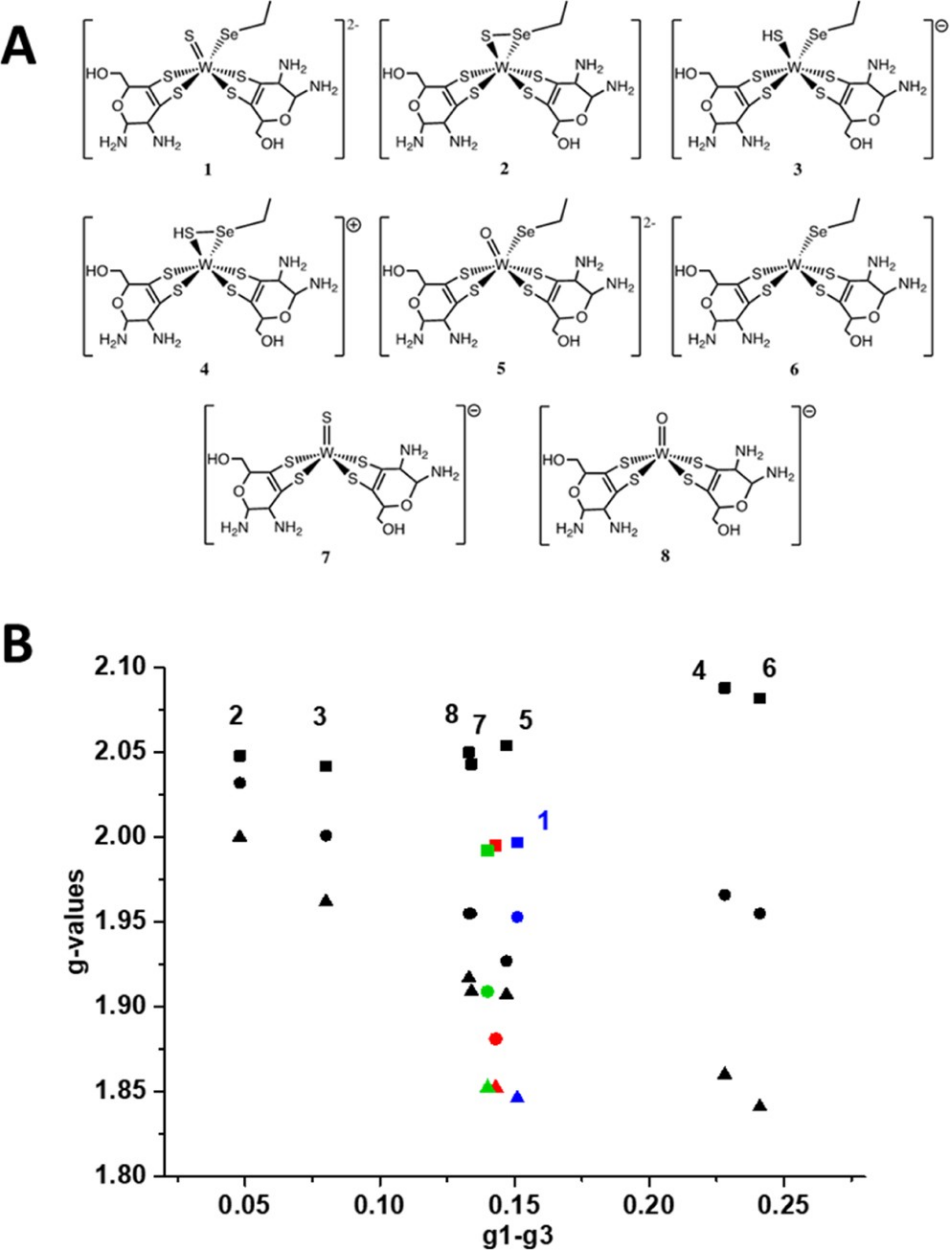
(A) Structural models of the W^V^ cofactor used for calculations. (B) Plot of the calculated *g*-values of the W^V^ species against anisotropy for the different models (indicated by numbers, model 1 in blue) and *g*-values of the experimental species W_D_^V^ (green) and W_F_^V^ (red symbols). *g*_1_, square; *g*_2_, circle; *g*_3_, triangle.

In order to correlate the structural properties of the W cofactor identified by crystallography and the EPR results, a quantum chemistry study was performed on several W^V^ cofactor models. Eight structural models (numbered **1** to **8**) were built based on the structure of the oxidized FdhAB.^8^ These models have in common the coordination of W^V^ by the four sulfur atoms of the two pterins, while they differ in the presence of the selenocysteine ligand or in the nature of the exogenous ligand (sulfur or oxygen), its protonation state, and the possible bond between the sulfur ligand and the selenium atom, as suggested for Mo coordination by S–S–Cys in periplasmic nitrate reductases (Figure 5A).^44,45^

It is well known that for Mo cofactors, *g*-value calculations are strongly affected by the “twist angle”, which corresponds to the SSSS dihedral angle between the two pyranopterins.^44^ It has been postulated that specific values of this angle could be related to the catalytic efficiency of the enzyme, a relationship that has been called the pterin twist hypothesis.^46,47^ This geometrical parameter is constrained by the protein environment of the Mo/W-bisMGD cofactor, and strikingly, in the crystal structures of FdhAB, it varies from −36° for the oxidized enzyme (PDB_code_6SDR) to −20° for the reduced enzyme (PDB_code_6SDV). Thus, for the eight structural models, constrained geometry optimizations were performed in which the twist angle was fixed to −28°, a value corresponding to the average between fully oxidized and fully reduced enzymes. The calculated *g*-values obtained for the different models were plotted as a function of the *g*-tensor anisotropy, a representation that is well adapted to analyze structural correlations between paramagnetic species,^5,23,44^ and compared to experimental parameters of the two W_D_^V^ and W_F_^V^ species (Figure 5B). Clearly, the best agreement between both *g*-values is obtained for model **1**, which corresponds to the crystal structures in oxidized and fully reduced states. The other models lead to markedly different anisotropies or mean values of the *g*-tensor.

Model **1** was then selected for refinement, and the dependence of *g*-values as a function of the twist angle was computed (Figure S4). This analysis shows that calculated *g*_1_ and *g*_3_ vary only slightly for SSSS dihedral angles ranging from −26 to −30° and are in excellent agreement with the experimental values determined for W_D_^V^ and W_F_^V^ species. In contrast, *g*_2_-values change significantly for the twist angles varying between −28 and −38°, and good agreement with *g*_2_ of W_D_^V^ and W_F_^V^ species is found for angle values of −33 and −35°, respectively (Figure S4). Moreover, although hyperfine coupling constant calculations are very sensitive to spin-density distribution which critically depends on geometry, A(^183^W) values were evaluated. Considering experimental uncertainties and the simplicity of model **1**, the values computed for a twist angle of −35° (A(^183^W)_1,2,3_ = 200, 125, and 75 MHz) are in reasonable agreement with the experimental ones (Table 2), thus supporting the validity of the model. Hence, model **1** accounts very satisfactorily for the FdhAB W^V^ cofactor *g*-values, and the main difference between W_D_^V^ and W_F_^V^ species might be a small variation of the SSSS dihedral angle. Such small conformational changes may result from distant structural influences induced either by the activation process or by the nature of the reductant. Indeed, it has been recently shown that in the closely related Mo-enzyme, respiratory nitrate reductase, some modifications of the H-bond network in the substrate access tunnel can remotely affect the spectroscopic and redox properties of the Mo cofactor.^48^ Although there is no structural difference in the substrate access tunnel in the fully reduced enzyme (W^IV^) either by dithionite or formate, similar effects are potentially at work in the intermediate W^V^ redox state of FdhAB in solution. The striking conversion of the W_F_^V^ species into W_D_^V^ upon dithionite addition might be explained by an indirect effect of dithionite on the H-bond network in the metal cofactor surrounding or in the substrate access tunnel.

Previous EPR characterization of the NAD^+^-dependent Mo-FDH from *Ralstonia eutropha* identified a strong proton hyperfine splitting of about 20 MHz in the Mo^V^ signal with a solvent-exchangeable site.^49^ This was considered to be the protonated sulfido group, consistent with the proposed reaction mechanism through direct hydride transfer.^49^ No similar proton hyperfine splitting was observed for the W^V^ EPR signals of FdhAB. Even if we cannot exclude that a proton hyperfine coupling is hidden by the larger line widths of W^V^ signals^32^ by comparison to Mo^V^, this result more likely reflects the absence of protonation of the sulfido group in the W^V^ species as deduced from the DFT analysis.

In conclusion, by combining EPR spectroscopy, X-ray crystallography, and DFT calculations, our results demonstrate that a six-ligand coordination of the W atom is maintained in the three redox states of the W cofactor, notably the binding of the Se atom. This contradicts the previously proposed catalytic mechanisms involving SeCys dissociation^43,50−52^ and clearly favors an outer sphere mechanism such as direct hydride transfer.^17,49,53^ The lack of proton hyperfine splitting of the W^V^ signals in FdhAB is not in contradiction with this latter mechanism since in the oxidative part of the catalytic cycle, a fast protonation step could follow the intermolecular electron transfer, leading to a W^V^=S intermediate instead of a W^V^–SH species (Figure S5). Pulsed EPR experiments will be helpful to investigate the H-bond network around the W cofactor and to clarify the possible differences between Mo and W FDHs, and studies are in progress toward this aim.

## Methods

### Protein Production

FdhAB was expressed and affinity-purified from *D. vulgaris* H, as previously described.^8^ Prior to EPR titrations, the buffer of pure FdhAB fractions was exchanged to 50 mM MOPS (3-(*N*-morpholino)propane sulfonic acid) buffer pH 7.6 with 10% (v/v) glycerol (buffer M) using a 30 kDa cutoff ultracentrifugation unit (Amicon Ultra-15 30 K NMWL, Millipore). Routine UV–visible absorption spectra were obtained with a Nanodrop ND2000C.

### Redox Titrations

FdhAB redox titrations were carried out inside a glovebox (Jacomex) at room temperature. Redox potentials were measured with a combined Pt–Ag/AgCl/KCl (3 M) microelectrode, in the presence of redox mediators (10 μM each): methylene blue (11 mV), indigo disulfonate (−125 mV), phenosafranine (−252 mV), methyl red (−325 mV), and methyl viologen (−440 mV). Before each titration, the electrode was calibrated using a redox buffer solution from Mettler-Toledo.

Titration using sodium dithionite was performed with FdhAB (70 μM) by stepwise addition of sodium dithionite solutions (≈60–240 mM in oxygen-free MOPS buffer). For titration with formate, FdhAB was treated with 50 mM DTT for 5–10 min and DTT was removed with buffer M using a 30 kDa cutoff ultracentrifugation unit, to a final concentration of 45 μM FdhAB. Stepwise additions of sodium formate solutions (≈20–100 mM) were carried out. After each addition, the potential was allowed to stabilize, and a sample was collected to an EPR tube and immediately frozen inside the glovebox using an ethanol bath refrigerated with liquid nitrogen from the outside. Frozen samples were transferred out of the glovebox and kept in liquid nitrogen. All potentials are against the standard hydrogen electrode.

Additionally, Ti(III)–citrate was freshly prepared as previously described^38,54^ and added stepwise to the as-isolated FdhAB (75 μM).

### EPR Spectroscopy

EPR experiments were performed on a Bruker ELEXSYS E500 spectrometer equipped with an ER4102ST standard rectangular Bruker EPR cavity fitted to an Oxford Instruments ESR 900 helium flow cryostat. Spin intensity measurements were performed by double integration of EPR spectra recorded under nonsaturating conditions and compared to a 1 mM Cu(II)EDTA standard. EPR spectrum simulations were performed with EasySpin.

### Crystallization, Data Collection, Structure Solution, and Refinement

The as-isolated FdhAB crystals were obtained using 26% PEG 3350, 0.1 M Tris-HCl pH 8.0, and 1 M LiCl, as reported.^8^ Crystals with 14 days old were soaked with 10 mM sodium dithionite for 10 min, before being transferred into a cryoprotectant solution consisting of the precipitant solution supplemented with 20% (v/v) glycerol, and then flash-cooled in liquid nitrogen. Details on data collection and processing are provided in the Supporting Information.

### Computational Calculations

All the calculations were performed at a DFT level of theory (see details in the Supporting Information).

## Supporting Information Available

Methods for X-ray data collection and processing, computational calculations, EPR spectra of W-FdhAB, redox potential dependence of FeS signals, calculated *g*-values for the computational model 1, proposed catalytic mechanism, crystallographic data processing and refinement statistics, and SSSS dihedral angle values for models and crystallographic structures (PDF)
