# Supplementary material for: Spectroscopic and Structural Characterization of Reduced Desulfovibrio vulgaris Hildenborough W-FdhAB Reveals Stable Metal Coordination during Catalysis
Source: ACS Chem Biol. 2022 Jun 29;17(7):1901–9. doi: 10.1021/acschembio.2c00336 (PMC9774666; doi:10.1021/acschembio.2c00336)
Supplement: Supplementary file 1 — cb2c00336_si_001.pdf [file cb2c00336_si_001.pdf]

Supporting Information for

Spectroscopic and structural characterization of reduced *D. vulgaris* Hildenborough W-FdhAB reveals stable metal coordination during catalysis

Ana Rita Oliveira[1], Cristiano Mota[2,3], Kateryna Klymanska[2,3], Frédéric Biaso[4], Maria João Romão[2,3*], Bruno Guigliarelli[4*], Inês Cardoso Pereira[1*]

[1]Instituto de Tecnologia Química e Biológica António Xavier, Universidade Nova de Lisboa, Av. da República, 2780-157 Oeiras, Portugal
[2]Associate Laboratory i4HB – Institute for Health and Bioeconomy, NOVA School of Science and Technology, Universidade NOVA de Lisboa, 2829-516 Caparica, Portugal
[3]UCIBIO, Applied Molecular Biosciences Unit, Departament of Chemistry, NOVA School of Science and Technology, Universidade NOVA de Lisboa, 2829-516 Caparica, Portugal
[4]Aix Marseille Univ, CNRS, BIP, Laboratoire de Bioénergétique et Ingénierie des Protéines, Marseille 13402, France

*Corresponding authors: ipereira@itqb.unl.pt, guigliar@imm.cnrs.fr and mjr@fct.unl.pt

Supplementary methods

X-ray data collection and processing

X-ray diffraction data were collected on XALOC beamline ate the ALBA synchrotron[1] and processed with the programs XDS[2], Pointless[3], and Aimless[4] as implemented in the autoPROC pipeline[5].

The structure of *Dv*FdhAB_dith was solved by molecular replacement with Phaser[6] from the CCP4 suite[7], using as search model the previously published formate-reduced structure (PDB_{code} 6SDV). The solution was improved with cycles of manual model building with Coot[8] and refinement with REFMAC5[9]. Data processing and refinement statistic are presented in Table S1.

Computational calculations

Geometry optimizations were carried out using the Gaussian16 software[10] with the B3LYP hybrid functional[11,12] and the def2svp basis set for all atoms[13,14]. For some models (indicated in the text), the bis-pyranopterin SSSS dihedral angle was kept fixed during optimization.

Electronic and magnetic properties were calculated on the optimized geometries using the quantum chemistry package Orca 4.2.1[15,16] with the B3LYP functional and the D3BJ dispersion correction[17–20]. Electronic structures were calculated using the segmented all-electron relativistically contracted (SARC) basis set of triple-ζ quality (TZVP) for tungsten and the def2-TZVPP basis set for the other atoms[14,18,19,21]. Relativistic effects were included using the zeroth-order regular approximation ZORA[22]. The spin-orbit mean-field Hamiltonian (SOMF) was used to account for the spin-orbit coupling.

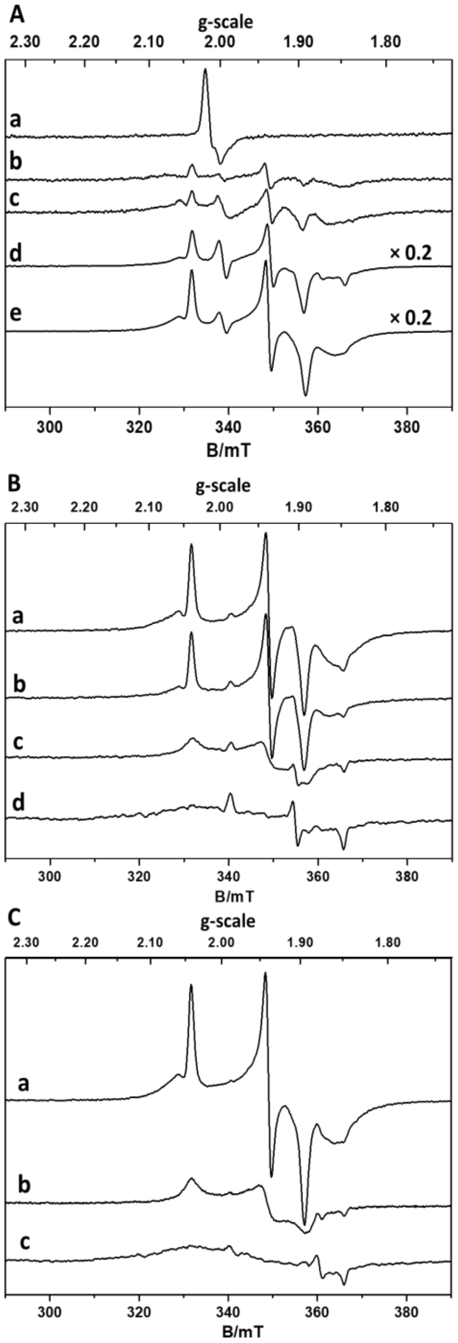

Figure S1 – EPR spectra of W-FdhAB and their temperature dependence. **A** - EPR spectra of FdhAB (a) as isolated 70 µM, *E* =+251 mV. The weak isotropic EPR signal centred at about g = 2.01 with asymmetric shape and fast relaxation properties is characteristic of an oxidized $[3Fe\text{-}4S]^{1+}$ cluster. Its spin intensity represents less than 0.03 spin/molecule indicating that this centre likely arises from a small degradation of the [4Fe-4S] centres of the enzyme; after dithionite reduction at -213 mV (b) and -472 mV (e); (c) after DTT treatment (45 µM, -161 mV); (d) after formate reduction of DTT treated enzyme (45 µM, - 483 mV) . Experimental conditions: temperature, 15 K, modulation amplitude, 1 mT at 100 kHz, microwave frequency, 9.4808 GHz; microwave power, 1 mW. **B** - Enzyme reduced by dithionite. Experimental conditions: temperature, 15 K (a), 20 K (b), 50 K (c), 80 K (d); Other conditions as in A, except microwave power, 1 mW (a, b) or 10 mW (c-d). **C** – Enzyme reduced with formate. Experimental conditions: temperature, 15 K (a), 50 K (b) 80 K (c). Other conditions as in A, except microwave power, 1 mW (a) or 10 mW (b and c). For signal to noise improvement, spectra A-d and B-c result from 10 scans accumulation and are divided by factor 5.

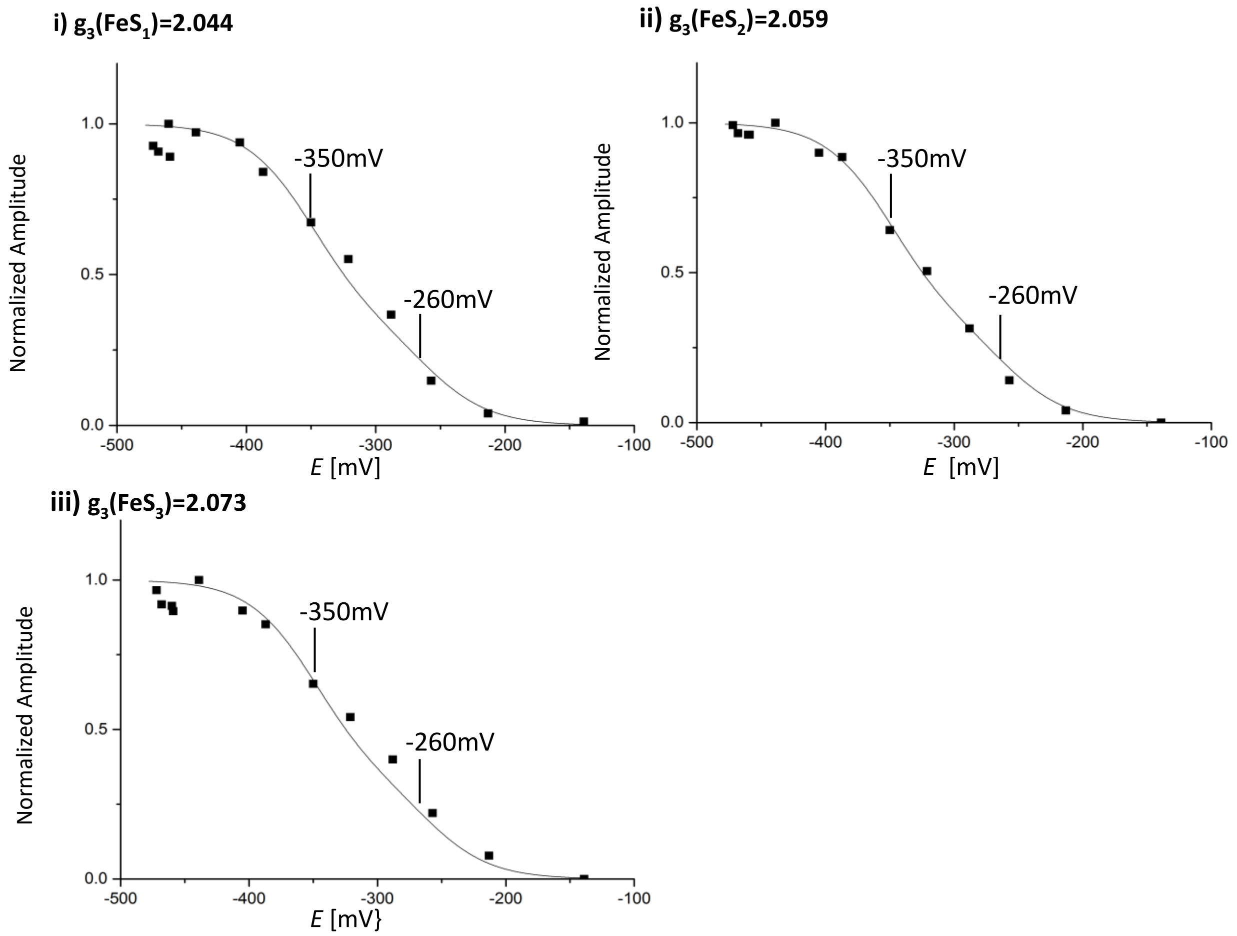

Figure S2 - Redox potential dependence of the FeS signals upon FdhAB titration with dithionite. EPR conditions as in Figure 3. Amplitude variation in i) g= 2.044, ii) g= 2.059, iii) g=2.073. The continuous line corresponds to the superimposition of two Nernst processes centered at E°1 = -260 mV and E°2 = -350 mV, with 1:2 relative contributions, respectively.

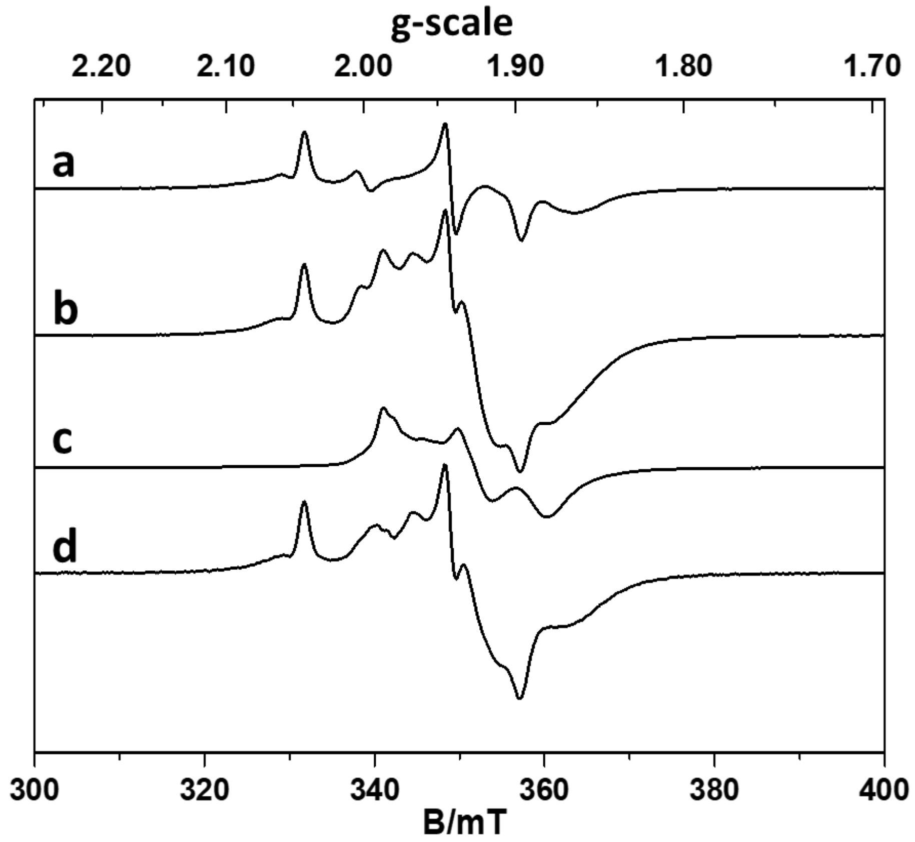

Figure S3: EPR spectra of Ti(III)-citrate reduced FdhAB. Samples poised at -420 mV (a) with substoichiometric amount of Ti(III)-citrate and at -525 mV (b, c) with an excess. d) is the spectral difference between b) and c). Experimental conditions: temperature 15 K (a, b), 80 K (c); microwave power 1mW (a, b); 40 mW (c); other conditions as in Fig. 1.

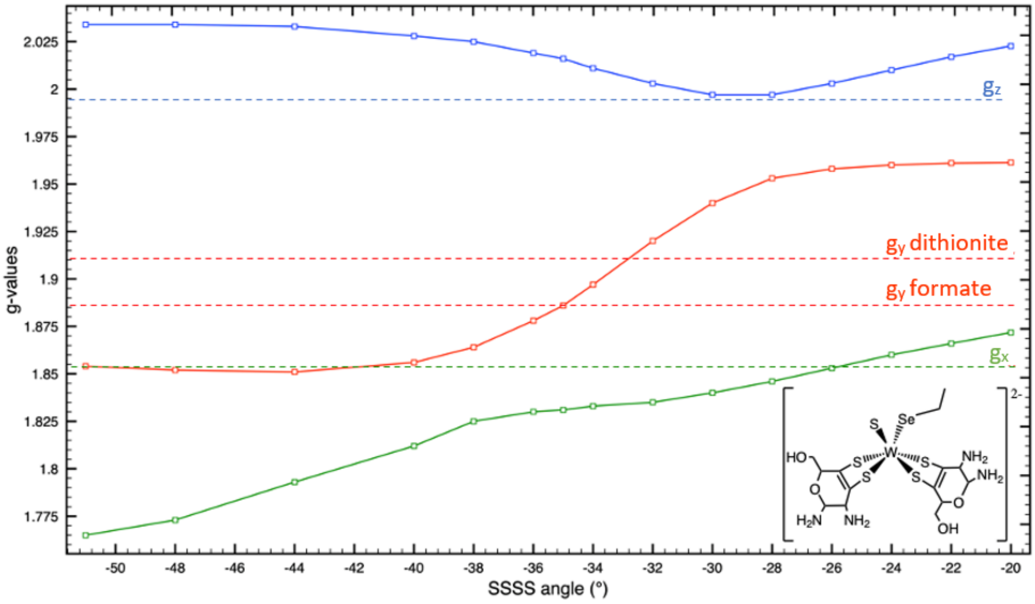

Figure S4 – Calculated g values for the computational model 1 versus the SSSS dihedral angle φ. The horizontal dashed lines indicate the experimental values for the W^{V} species.

For all models (except model 3), full optimization leads to conformations with values of φ far from those obtained in crystal structures (Table S2). This is in agreement with the pterin twist hypothesis recently formulated by Warelow et al.[23], which postulated that protein constraints the bis-pyranopterin cofactor to favor a geometry close to the transition state.

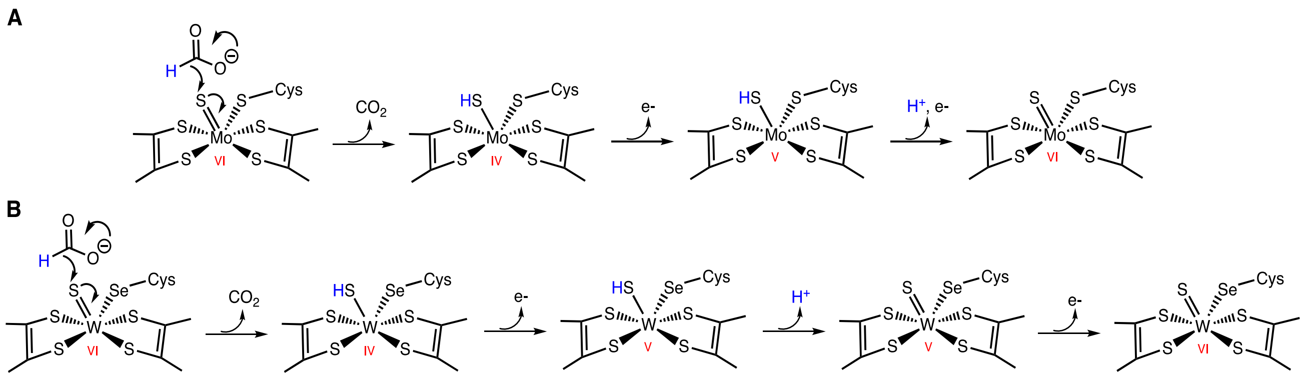

Figure S5 – (A) Proposed catalytic mechanism for *Cupriavidus necator* Mo- Formate dehydrogenase (adapted from Yu et al.[24]). (B) Proposed catalytic mechanism for *Desulfovibrio vulgaris* Hidenborough W-FdhAB.

Table S1 - Crystallographic data processing and refinement statistics

**Diffraction Data – *Dv*FdhAB_dith**		**Geometry and B-factor**	
Space Group	$P2_12_12_1$	RMSD bond lengths (Å)	0.001
Cell Dimensions (Å)	a=64.8, b=124.1, c=149.6.	RMSD bond angles (°)	1.473
		RMS chirality (Å^3)	0.107
		B-factor All (Å^2)	22.22
Wavelength (Å)	0.979	B-factor Protein (Å^2)	23.68
Energy (keV)	12.66	B-factor Ligands (Å^2)	
Crystal – detector distance (mm)	274.77	MGD	14.74
Oscillation angle (°)	0.1	[4Fe-4S]	15.45
Beamline	BL13-XALOC (ALBA)	W	14.07
		S	16.73
Resolution range (Å)	95.55-1.53 (1.55-1.53)	GOL	40.84
		PEG	47.88
Completeness (%)	99.6 (99.7)	NO_3	57.99
Solvent content (%)	47.00	Solvent (Å^2)	33.78
Protein molecules per asymmetric unit	1	Ramachandran favoured (%)	97.00
Matthews coefficient (Å^3.Da^{-1})	2.32	Ramachandran outliers (%)	0.17
R_{merge}	0.090 (1.072)	Ramachandran allowed (%)	2.83
R_{means}	0.102 (1.212)	Rama distribution Z-score	-0.84 ± 0.23
I/σI	9.8 (1.5)	Molprobity score	1.48
Multiplicity	4.5 (4.6)	Clashscore	2.92
Total reflections	821848 (42034)		
Unique reflections	182484 (9070)		
CC (1/2)	0.997 (0.591)		
**Refinement statistics**			
R_{work}	0.165		
R_{free}	0.192		
Number of atoms			
Protein	9197		
Ligands	197		
Solvent	857		

Table S2 – SSSS dihedral angle values φ for fully optimized models and crystallographic structures

**Model**	1	2	3	4	5	6	7	8	6SDR	6SDV
**φ (°)**	-51	-47	-35	-5	-52	-2	0	0	-36	-20

References

(1) Juanhuix, J.; Gil-Ortiz, F.; Cuní, G.; Colldelram, C.; Nicolás, J.; Lidón, J.; Boter, E.; Ruget, C.; Ferrer, S.; Benach, J. Developments in Optics and Performance at BL13-XALOC, the Macromolecular Crystallography Beamline at the Alba Synchrotron. *J. Synchrotron Radiat.* **2014**, *21* (4), 679–689. https://doi.org/10.1107/S160057751400825X.

(2) Kabsch, W. XDS. *Acta Crystallogr. Sect. D Biol. Crystallogr.* **2010**, 125–132. https://doi.org/10.1107/S0907444909047337.

(3) Evans, P. Scaling and Assessment of Data Quality. *Acta Crystallogr. Sect. D Biol. Crystallogr.* **2006**, *62* (1), 72–82. https://doi.org/10.1107/S0907444905036693.

(4) Evans, P. R.; Murshudov, G. N. How Good Are My Data and What Is the Resolution? *Acta Crystallogr. Sect. D Biol. Crystallogr.* **2013**, *69* (7), 1204–1214. https://doi.org/10.1107/S0907444913000061.

(5) Vonrhein, C.; Flensburg, C.; Keller, P.; Sharff, A.; Smart, O.; Paciorek, W.; Womack, T.; Bricogne, G. Data Processing and Analysis with the AutoPROC Toolbox. *Acta Crystallogr. Sect. D Biol. Crystallogr.* **2011**, *67* (4), 293–302. https://doi.org/10.1107/S0907444911007773.

(6) McCoy, A. J.; Grosse-Kunstleve, R. W.; Adams, P. D.; Winn, M. D.; Storoni, L. C.; Read, R. J. Phaser Crystallographic Software. *J. Appl. Crystallogr.* **2007**, *40* (4), 658–674. https://doi.org/10.1107/S0021889807021206.

(7) Winn, M. D.; Ballard, C. C.; Cowtan, K. D.; Dodson, E. J.; Emsley, P.; Evans, P. R.; Keegan, R. M.; Krissinel, E. B.; Leslie, A. G. W.; McCoy, A.; McNicholas, S. J.; Murshudov, G. N.; Pannu, N. S.; Potterton, E. A.; Powell, H. R.; Read, R. J.; Vagin, A.; Wilson, K. S. Overview of the CCP4 Suite and Current Developments. *Acta Crystallogr. Sect. D Biol. Crystallogr.* **2011**, *67* (4), 235–242. https://doi.org/10.1107/S0907444910045749.

(8) Emsley, P.; Lohkamp, B.; Scott, W. G.; Cowtan, K. Features and Development of Coot. *Acta Crystallogr. Sect. D Biol. Crystallogr.* **2010**, *66* (4), 486–501. https://doi.org/10.1107/S0907444910007493.

(9) Murshudov, G. N.; Skubák, P.; Lebedev, A. A.; Pannu, N. S.; Steiner, R. A.; Nicholls, R. A.; Winn, M. D.; Long, F.; Vagin, A. A. REFMAC5 for the Refinement of Macromolecular Crystal Structures. *Acta Crystallogr. Sect. D Biol. Crystallogr.* **2011**, *67* (4), 355–367. https://doi.org/10.1107/S0907444911001314.

(10) Frisch, M. J.; Trucks, G. W.; Schlegel, H. B.; Scuseria, G. E.; Robb, M. A.; Cheeseman, J. R.; Scalmani, G.; Barone, G. A.; Petersson, G. A.; Nakatsuji, H.; Li, X.; Caricato, M.; Marenich, A. V.; Bloino, J.; Janesko, B. G.; Gomperts, R.; Mennucci, B.; Hratchian, H. P.; Ortiz, J. V.; Izmaylov, A. F.; Sonnenberg, J. L.; Williams-Young, D.; Ding, F.; Lipparini, F.; Egidi, F.; Goings, J.; Peng, B.; Petrone, A.; Henderson, T.; Ranasinghe, D.; Zakrzewski, V. G.; Gao, J.; Rega, N.; Zheng, G.; Liang, W.; Hada, M.; Ehara, M.; Toyota, K.; Fukuda, R.; Hasegawa, J.; Ishida, M.; Nakajima, T.; Honda, Y.; Kitao, O.; Nakai, H.; Vreven, T.; Throssell, K.; Montgomery Jr., J. A.; Peralta, J. E.; Ogliaro, F.; Bearpark, M. J.; Heyd, J. J.; Brothers, E. N.; Kudin, K. N.; Staroverov, V. N.; Keith, T. A.; Kobayashi, R.; Normand, J.; Raghavachari, K.; Rendell, A. P.; Burant, J. C.; Iyengar, S. S.; Tomasi, J.; Cossi, M.; Millam, J. M.; Klene, M.; Adamo, C.; Cammi, R.; Ochterski, J. W.; Martin, R. L.; Morokuma, K.; Farkas, O.; Foresman, J. B.; Fox, D. J. Gaussian. *Inc., Wallingford CT,* **2016**.

(11) Becke, A. D. Density-functional Thermochemistry. III. The Role of Exact Exchange. *J. Chem. Phys.* **1993**, *98* (7), 5648–5652. https://doi.org/10.1063/1.464913.

(12) Lee, C.; Yang, W.; Parr, R. G. Development of the Colle-Salvetti Correlation-Energy Formula into a Functional of the Electron Density. *Phys. Rev. B* **1988**, *37* (2), 785–789. https://doi.org/10.1103/PhysRevB.37.785.

(13) Andrae, D.; Häußermann, U.; Dolg, M.; Stoll, H.; Preuß, H. Energy-Adjusted Ab Initio Pseudopotentials for the Second and Third Row Transition Elements. *Theor. Chim. Acta* **1990**, *77* (2), 123–141. https://doi.org/10.1007/BF01114537.

(14) Weigend, F.; Ahlrichs, R. Balanced Basis Sets of Split Valence, Triple Zeta Valence and Quadruple Zeta Valence Quality for H to Rn: Design and Assessment of Accuracy. *Phys. Chem. Chem. Phys.* **2005**, *7* (18), 3297. https://doi.org/10.1039/b508541a.

(15) Neese, F. The ORCA Program System. *WIREs Comput. Mol. Sci.* **2012**, *2* (1), 73–78.

https://doi.org/10.1002/wcms.81.
(16) Neese, F. Software Update: The ORCA Program System, Version 4.0. *WIREs Comput. Mol. Sci.* **2018**, *8* (1), 1–6. https://doi.org/10.1002/wcms.1327.
(17) Grimme, S. Accurate Description of van Der Waals Complexes by Density Functional Theory Including Empirical Corrections. *J. Comput. Chem.* **2004**, *25* (12), 1463–1473. https://doi.org/10.1002/jcc.20078.
(18) Grimme, S.; Antony, J.; Ehrlich, S.; Krieg, H. A Consistent and Accurate Ab Initio Parametrization of Density Functional Dispersion Correction (DFT-D) for the 94 Elements H-Pu. *J. Chem. Phys.* **2010**, *132* (15), 154104. https://doi.org/10.1063/1.3382344.
(19) Grimme, S.; Ehrlich, S.; Goerigk, L. Effect of the Damping Function in Dispersion Corrected Density Functional Theory. *J. Comput. Chem.* **2011**, *32* (7), 1456–1465. https://doi.org/10.1002/jcc.21759.
(20) Grimme, S. Semiempirical GGA-Type Density Functional Constructed with a Long-Range Dispersion Correction. *J. Comput. Chem.* **2006**, *27* (15), 1787–1799. https://doi.org/10.1002/jcc.20495.
(21) Pantazis, D. A.; Chen, X. Y.; Landis, C. R.; Neese, F. All-Electron Scalar Relativistic Basis Sets for Third-Row Transition Metal Atoms. *J. Chem. Theory Comput.* **2008**, *4* (6), 908–919. https://doi.org/10.1021/ct800047t.
(22) Van Wüllen, C. Molecular Density Functional Calculations in the Regular Relativistic Approximation: Method, Application to Coinage Metal Diatomics, Hydrides, Fluorides and Chlorides, and Comparison with First-Order Relativistic Calculations. *J. Chem. Phys.* **1998**, *109* (2), 392–399. https://doi.org/10.1063/1.476576.
(23) T.P Warelow, M. J. Pushie, J.J.H. Cotelesage, J.M. Santini, G.N.George. *Scientific Rep.* **2017**, *7*, 1757. DOI : 10.1038/s41598-017-01840-y
(24) Yu X., Niks D., Mulchandani A., Hille R. Efficient reduction of CO_2 by the molybdenum-containing formate dehydrogenase from *Cupriavidus necator* (*Ralstonia eutropha*). *J Biol Chem.* **2017,** 292, 16872-16879. doi: 10.1074/jbc.M117.785576.
